# Comparing ChatGPT-3.5, Gemini 2.0, and DeepSeek V3 for pediatric pneumonia learning in medical students

**DOI:** 10.1038/s41598-025-27722-2

**Published:** 2025-11-18

**Authors:** Kubra Aykac, Osman Cubuk, Osman Oguz Demir, Young June Choe, Malik Aydin, Yasemin Ozsurekci

**Affiliations:** 1 Department of Pediatric Infectious Diseases, Hacettepe University, 06100 Ankara, Turkey; 2 Department of Pediatrics, Korea University Anam Hospital, Seoul, 02841 Korea; 3 Laboratory of Translational Medicine and Pediatric Infectious Diseases, Center for Biomedical Education and Research (ZBAF), Faculty of Health, Department of Human Medicine, Witten/Herdecke University, Witten, Germany; 4 Virology and Microbiology, Faculty of Health, Center for Biomedical Education and Research, School of Life Sciences (ZBAF), Witten/Herdecke University, Stockumerstr. 10, 54435 Witten, Germany; 5 Chair of Pediatrics, Vestische Kinder- und Jugendklinik Datteln, Witten/Herdecke University, 45711 Datteln, Germany

**Keywords:** Artificial intelligence, Medical education, Pneumonia, Lower respiratory tract infection, Children, Computational biology and bioinformatics, Diseases, Health care, Medical research

## Abstract

Pediatric pneumonia (PP) remains an important topic in undergraduate medical education and offers a suitable framework for evaluating large language models (LLMs) in AI-assisted learning. We developed a 27 open-ended survey including five core domains of PP, such as diagnosis, etiology, diagnostics, treatment, and prevention. DeepSeek V3, Gemini 2.0, and ChatGPT-3.5, were each provided with identical reference materials. Two pediatric infectious disease specialists independently assessed responses using a structured 10-point rubric through Licert, which presents a custom evaluation tool. DeepSeek V3 achieved the highest mean score (9.9), outperforming ChatGPT-3.5 (7.7), and Gemini 2.0 (7.5) through all domains (*p* < 0.001). Moreover, it received a full score in 26 out of 27 questions (96.3%) and achieved an accuracy score of  ≥ 5. In addition, the highest performance appeared in higher-order reasoning areas, including age-specific etiology and imaging interpretation, with DeepSeek V3 outperforming others by up to 3.2 points. While all models demonstrated almost safety, the variability in content quality however highlights the necessity for careful platform selection. Therefore, future research should explore educational outcomes comparing AI-assisted and conventional learning approaches to better define the role of LLMs in medical education.

**Supplementary Information:**

The online version contains supplementary material available at 10.1038/s41598-025-27722-2.

## Introduction

The integration of artificial intelligence (AI) tools, in particular large language models (LLMs), in medical education is increasing. This expansion has generated both enthusiasm and critical reflection among students, teachers, scholars, and clinicians.^1^^,^^2^^,^^3^ A remarkable situation during a clinical oral examination at our university exemplified this trend, where a medical student described the vesicular rash of varicella (also termed as chickenpox) as a ‘starry sky’, which were an unfamiliar and metaphorical phrase to both examiners and was not stated within the current textbooks.^4^^,^^5^ Upon further discussion, the student revealed that this terminology was learned through ChatGPT-3.5. This moment highlighted not only the expanding role of LLMs in the learning processes of medical students, but also the potential influence of these tools on the clinical vocabulary and diagnostic work-up among students.

Influenced by this observation, the current literature was then studied and revealed an increase amount of research exploring the capabilities and limitations of LLMs in medical education and clinical support.^6^^,^^7^ Most studies have primarily addressed the diagnostic accuracy with clinical guidelines. However, only few focused on the educational use of these models in teaching specific main topics.^7^^,^^8^^,^^9^

Pediatric pneumonia (PP) remains one of the most important and critical topics in undergraduate medical training. It is a leading cause of morbidity and mortality among children under five years of age worldwide, in particular in low- and middle-income countries.^10^ Its understanding on clinical presentation, diagnostic workup, and management requires integration of knowledge from infectious diseases, respiratory medicine, radiology, and pharmacology. The complexity and prevalence of PP may also present a relevant subject to evaluate the educational potential of LLMs for medical students.

To address this, we therefore designed the present study to systematically evaluate the performance of three currently known LLMs, including ChatGPT-3.5 (OpenAI), Gemini 2.0 (Google), and DeepSeek V3 in responding to clinically relevant and educationally oriented questions about PP. Our primary objective was to determine, which model may provide the most accurate and comprehensive tool, guiding professors, scholars, and educators, as well as students in selecting the most effective AI-based learning platform for this key topic in pediatric infectious diseases.

## Material and method

### Study design and objective

This is a cross-sectional and comparative evaluation study designed to investigate the performance of three LLMs, including ChatGPT-3.5 (OpenAI)**,** Gemini 2.0 (Google), and DeepSeek V3 in providing educational content focusing on PP**.** The primary objective is to determine the accuracy, completeness, and safety of these models to questions, which are commonly used by medical students during their medical training. To simulate a controlled learning environment, we provided each model with the full text of the Community-Acquired Pneumonia chapter from ‘Nelson Textbook of Pediatrics’^11^, the ‘Revised WHO classification and treatment of childhood pneumonia at health facilities publication’^12^, and the 2011 IDSA guideline^13^ for the management of community-acquired pneumonia in children. These resources represent as the most relevant reference materials available to the models. This study did not involve any human participants, including medical students. All analyses were performed using model-generated outputs. An ethics or institutional review board approval was thus not necessary.

### Question design and content domains

A total of 27 open-ended questions were created by pediatric infectious disease specialists, focusing on highly relevant topics related to PP. These questions were classified into five different clinical domains of pneumonia (Supplementary Table 1):

1. Diagnosis and Clinical Features (Q1 to Q5)
2. Etiology and Age-Specific Pathogens (Q6 to Q11)
3. Diagnostics and Imaging (Q12 to Q16)
4. Complications (Q17 to Q18)
5. Management, Treatment, and Prevention (Q19 to Q27)

All questions correlated to commonly tested knowledge fields and clinical reasoning skills expected from medical students in their pediatric trainee rotations.

### Model interaction and data collection

Each question was submitted to the three LLMs (ChatGPT-3.5, Gemini 2.0, and DeepSeek V3) under standardized prompting, without any follow-up clarification or refinement, and responses were then collected.

### Evaluation criteria

The evaluation process was performed using Language Intelligence Certifier (Licert), which is a custom-based tool developed for structured scoring and performance assessment of AI-generated responses. Each model response was independently evaluated by two pediatric infectious disease specialists, who were blinded to model identity. Their evaluations followed a structured rubric addressing specific dimensions:

#### Accuracy, using a 6-point Likert scale (1–6 points)

i. 1 = completely incorrect and 6 = completely correct: Clinical correctness and factual precision.

#### Completeness, using a 3-point Likert scale (1–3 points)

i. 1 = incomplete and 3 = comprehensive: Inclusion of key concepts and breadth of coverage.

#### Safety, using a binary score (0–1 point)

i. 0 = clinically unsafe and 1 = clinically safe: Appropriateness and avoidance of harmful or misleading content.

#### The total score ranged from 2 to 10 points per question.

The responses rated as completely incorrect (accuracy = 1) were excluded from the completeness evaluation. Furthermore, the disagreements between reviewers were solved by consensus or by averaged scores.

### Statistical analysis

All analyses were performed using R (4.1.1, The R Foundation for Statistical Computing, Vienna, Austria). The scores were summarized by individual questions and clinical domains. The Friedman test was used to address overall differences among models, followed by post-hoc Wilcoxon signed-rank tests for pairwise comparisons. In addition, the Benjamini–Hochberg correction was performed to adjust for multiple testing. The results are presented as mean ± standard error of the mean (SEM), and statistical significance was set at *p* < 0.05. Significant pairwise differences are indicated in the figure with brackets and adjusted *p*-values.

## Results

A total of 27 clinically relevant questions related to PP were used to three LLMs. The answers of each AI model was evaluated using a structured scoring system based on three domains, including accuracy (scored 1 to 6), completeness (scored 1 to 3), and clinical safety (scored 0 to 1), yielding a maximum score of 10 per response, across all questions, which resulted in 81 individual responses and 243 separate evaluator judgments (Supplementary Table 2).

### Overall model performance

Across all 27 questions on PP, DeepSeek V3 outperformed ChatGPT-3.5 and Gemini 2.0 (Table 1). It showed the highest mean total score per question (9.9, range: 9.8–10) across domains, followed by ChatGPT-3.5 (7.7, range: 6.8–10) and Gemini 2.0, which achieved a score of 7.5 (range: 6.8–8.5) (Table 2). DeepSeek V3 resulted into the highest score in 26 out of 27 questions (96.3%), which shared the top position with other models in five questions, twice with ChatGPT-3.5, once with Gemini 2.0, and twice with ChatGPT-3.5 and Gemini 2.0.

**Table 1:** Performance scores of ChatGPT-3.5, Gemini 2.0, and DeepSeek V3 across 27 questions on pediatric pneumonia. The ‘Best Performing Model’ column indicates the highest-scoring AI for each question.

Question No	ChatGPT-3.5	Gemini 2.0	DeepSeek V3	Best performing model
Q1	8	8	10	DeepSeek V3
Q2	7	7	10	DeepSeek V3
Q3	8	8	10	DeepSeek V3
Q4	8	8	10	DeepSeek V3
Q5	6	6	10	DeepSeek V3
Q6	7	7	10	DeepSeek V3
Q7	6	6	10	DeepSeek V3
Q8	8	8	10	DeepSeek V3
Q9	6	6	10	DeepSeek V3
Q10	7	7	10	DeepSeek V3
Q11	7	7	10	DeepSeek V3
Q12	10	8	10	ChatGPT-3.5 and DeepSeek V3
Q13	6	6	10	DeepSeek V3
Q14	8	8	10	DeepSeek V3
Q15	6	6	10	DeepSeek V3
Q16	6	6	10	DeepSeek V3
Q17	10	7	10	ChatGPT-3.5 and DeepSeek V3
Q18	10	10	10	ChatGPT-3.5 , Gemini 2.0, and DeepSeek V3
Q19	6	6	10	DeepSeek V3
Q20	1	10	10	DeepSeek V3
Q21	7	7	10	DeepSeek V3
Q22	9	10	9	Gemini 2.0
Q23	8	10	10	Gemini 2.0 and DeepSeek V3
Q24	9	6	10	DeepSeek V3
Q25	10	10	10	ChatGPT-3.5, Gemini 2.0, and DeepSeek V3
Q26	6	6	10	DeepSeek V3
Q27	7	6	10	DeepSeek V3

**Table 2:** Mean performance scores by clinical domain, demonstrating highest scores of DeepSeek V3  across all categories (i) Diagnosis, ii) Etiology, iii) Diagnostics, iv) Complications, and v) Management).

Category	ChatGPT-3.5 (Mean)	Gemini 2.0 (Mean)	DeepSeek V3 (Mean)	Top performer
Diagnosis and Clinical Features (Q1-5)	7.4	7.4	10	DeepSeek V3
Etiology and Age-Specific Agents (Q6-11)	6.8	6.8	10	DeepSeek V3
Diagnostics and Imaging (Q12-16)	7.2	6.8	10	DeepSeek V3
Complications (Q17-18)	10	8.5	10	DeepSeek V3
Management, Treatment, and Prevention (Q19-27)	7	7.8	9.8	DeepSeek V3

### Domain-specific performance

We then analyzed the clinical domains for following aspects:

- *Diagnosis and Clinical Features (Q1–Q5)*: DeepSeek V3 scored a perfect range of 10, outperforming both ChatGPT-3.5 and Gemini 2.0, which scored each 7.4.
- *Etiology and Age-Specific Agents (Q6–Q11)*: DeepSeek V3 also achieved a perfect score of 10, while ChatGPT-3.5 and Gemini 2.0 both scored 6.8, where this domain, along with ‘Diagnostics and Imaging’, showed the widest performance gap between the models (3.2 points).
- *Diagnostics and Imaging (Q12–Q16)*: DeepSeek V3 led with a perfect score of 10, whereas ChatGPT-3.5 slightly outperformed Gemini 2.0 (7.2 vs. 6.8). Similar to ‘Etiology’, this domain showed a 3.2 point difference between the highest and lowest scoring models.
- *Complications (Q17–Q18)*: Both DeepSeek V3 and ChatGPT-3.5 received also perfect scores of 10, while Gemini 2.0 scored slightly lower at 8.5. This domain showed relatively minor variation among models.
- *Management, Treatment, and Prevention (Q19–Q27)*: DeepSeek V3 resulted a high mean score of 9.8, and Gemini 2.0 (7.8) outperformed ChatGPT-3.5 (7.0), indicating moderate variation across models in this domain.

These trends indicate that DeepSeek V3 was the most comprehensive and accurate tool across both knowledge and safety-related questions (Table 1 and Table 2).

### Evaluation criteria breakdown

**Fig. 1:**
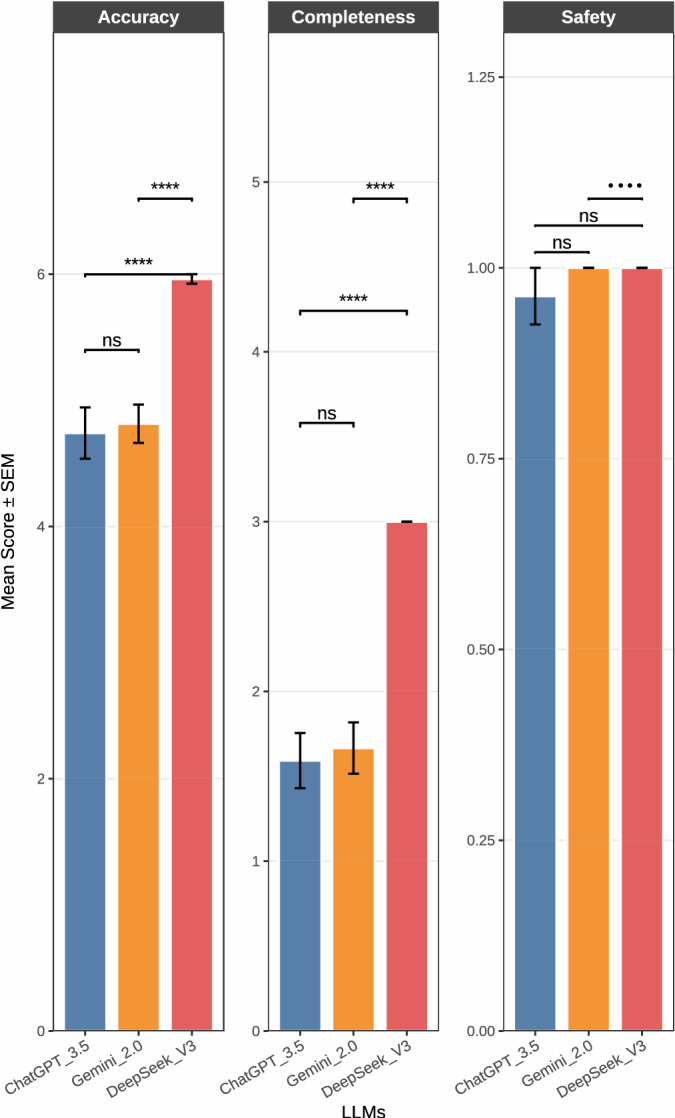
Mean accuracy, completeness, and safety scores of ChatGPT-3.5, Gemini 2.0, and DeepSeek V3 across 27 pediatric pneumonia questions. The bars represent average scores of each evaluation domain. DeepSeek V3 demonstrated the highest mean scores in all three categories, with particularly notable differences in accuracy and completeness. All models showed high safety scores. The significance levels were determined using the Friedman test followed by Wilcoxon signed-rank pairwise comparisons, with *p*-values adjusted through the Benjamini–Hochberg method.

Figure 1 summarizes comparative model performances across three criteria. Here, DeepSeek V3 had the highest mean accuracy scores across all questions (*p* < 0.001), followed by the completeness, where DeepSeek V3 provided the most content-rich responses (*p* < 0.001). All three AI models performed similarly, none of them demonstrated unsafe or critical content in any model response. Statistical analysis using Friedman test followed by Wilcoxon signed-rank tests confirmed significant differences in performance towards models for both accuracy and completeness, with DeepSeek V3 significantly outperforming the others (*p*-adjusted < 0.05 using Benjamini–Hochberg correction).

### Safety and accuracy

Only one response for Q20 using ChatGPT-3.5 resulted as potentially unsafe due to clinically inaccurate information, whereas DeepSeek V3 and Gemini 2.0 provided the highest safety score , achieving a score of 1 (safe) in all 27 questions, making also this AI platform as the most promising tool for medical students in the context of PP (Supplementary Table 2). Furthermore, DeepSeek V3 revealed no responses with accuracy scores below than 4, indicating that this consistently may deliver answers that were more correct than incorrect. In contrast, ChatGPT-3.5 and Gemini 2.0 had multiple responses rated as clinically inaccurate (accuracy score < 4), without safety compromise.

## Discussion

This study provides a comparative analysis of three LLMs, including ChatGPT-3.5, Gemini 2.0, and DeepSeek V3 in the context of education on PP for medical students. Among the models evaluated, DeepSeek V3 demonstrated superior overall performance**,** demonstrating more accurate, comprehensive, and educationally sound responses across all clinical domains. These results also underscore the increasing potential of AI-based tools as supplementary learning materials in medical education, while also highlighting the necessity for critical evaluation of model quality before integration into the medical curriculum.^14–17^ Previous studies have mainly focused on general clinical accuracy of ChatGPT or its use in simulated board exams.^18^^,^^19^ To the best of our knowledge, our study is among the first to directly compare three competitive LLMs using a clinically targeted question set that reflects the educational requirements of medical students in a specific pediatric disease context. PP was chosen due to its high prevalence**,** clinical complexity, and relevance in medical training.^12^ According to the World Health Organization, pneumonia has still the highest mortality rate among children under five years of age, which is responsible for approximately 740,000 deaths in 2019.^10^ The selected domains ranging from diagnosis to prevention represent core competencies expected of any graduating medical student.

The consistent superiority of DeepSeek, in particular in the domains of diagnostic reasoning and treatment selection, may be highly relevant to more optimized medical datasets or better for educational content.^20^^,^^21^ In contrast, while ChatGPT-3.5 and Gemini 2.0 performed adequately in terms of safety and basic correctness, they also lacking the completeness or clinical depth, which are relevant and requiring for robust student learning. However, all models avoided critical or misleading content in terms of PP, suggesting that recent safety tools are potentially working effectively,^22^ even when the knowledge varies differentially. Of note, Q22 was the only item, where DeepSeek V3 did not achieve the highest score. Although all three models provided guideline-consistent answers, their usability for undergraduate learners also differed. DeepSeek V3 showed a highly detailed response, including dosing regimens and management in neonatal or immunocompromised cases. In contrast, more balanced response of Gemini 2.0 focusing on the core therapeutic approach and clinical decision points was evaluated on an educational appropriateness at this point.

One unexpected finding was the performance gap in higher-order domains, such as diagnostic age-specific etiologic considerations and imaging interpretation, where DeepSeek V3 maintained high scores compared to other AI models. This highlights a potential opportunity for future model training efforts for medical students focusing on domain-specific differences and nuances, especially in pediatrics, where age-specific background information and knowledge are highly relevant.^23^

**Fig. 2:**
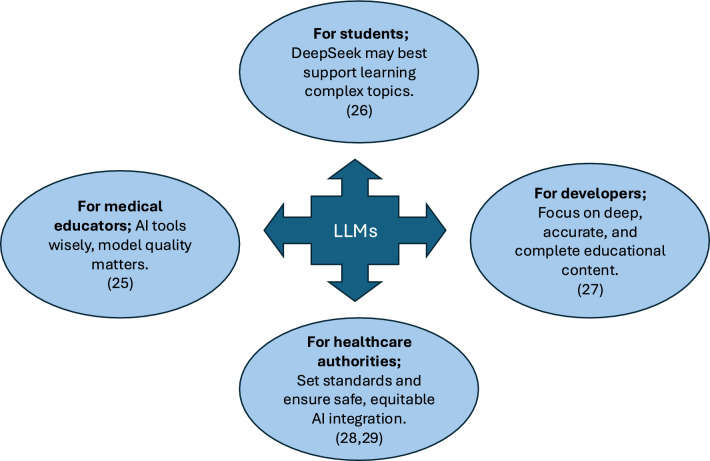
Summary of specific implications for integrating large language models (LLMs) into pediatric medical education. This figure highlights key responsibilities and considerations for medical educators, students, AI developers, and healthcare authorities in the ethical and effective implementation of AI-driven educational tools.^25-29^

Our observations might have several implications (Fig. 2). For medical educators, they may show that LLMs do not perform at the same level, so choosing the right platform is important when guiding students.^24^ For students, models such as DeepSeek V3 may be more safe when studying complex clinically topics.^25^ For AI developers, the priority should extend beyond basic accuracy to include completeness and subject-specific depth in educational content.^26^ Finally, for healthcare authorities, it will be essential to establish standards and ethical guidelines for the use of LLMs in medical education.

As AI continues to support and influence daily life, future studies should be expanded beyond single-disease evaluations to include integrated case-based learning**,** and interactive clinical simulations. The transformative potential of LLMs in enhancing medical education outcomes and learner engagement might be promising.^15^ Li and Wu (2025) described that these tools may help generate clinical content, support problem-based learning, and improve academic performance among medical students.^6^ At the same time, they can also point out critical concerns including automation bias and inaccuracies.^6^ Longitudinal studies examining the role of LLM-assisted learning on student performance and knowledge are also important. Furthermore, with the development of new models and the continuous updating of existing ones, it will be important to perform regular reviews (or analysis) to enable AI tools remaining consistent with clinical guidelines and educational standards. Although LLMs have shown promising outcomes, their accuracy also still varies widely. We therefore need more research on whether AI methods work reliably before implementing into real-world infectious disease care.^29^ Collaborations between educators, clinicians, developers, and even healthcare authorities will be highly relevant to redesign and improve LLMs not just as information sources, but as interactive, context-aware learning platforms. Our findings thus highlight the need to optimize these models for domains that require age-specific knowledge, such as pediatric diseases. Although LLMs can provide valuable support to physicians in selected tasks, they also carry important risks, as they may generate inaccurate information, overlook critical clinical context, and function as “black boxes” that can reinforce existing biases. Due to these limitations, they cannot be used on their own for infectious disease consultations, and specialists need to be involved to make sure they are used safely.^30^

Despite the promising results, this study should be interpreted within its limitations. First, the questions were limited to a single disease area, which may not fully represent model performance across all pediatric topics. Second, although human expert scoring was used, some subjectivity remains in rubric-based evaluations. Furthermore, a key limitation is the rapid LLM development, including the versions evaluated in this study (ChatGPT-3.5, Gemini 2.0, and DeepSeek V3) were current at the time of analysis, however newer versions have been since released. Considering these information, our findings thus reflect a brief overview of performance at a specific point in time and are not intended as definitive observations. Therefore, future studies using newer models should focus more on the methodology of structured evaluation and the interpretation of model outputs, rather than comparisons between specific versions.

## Conclusion

In this comparative evaluation of three LLMs, DeepSeek V3 presented as the most effective AI tool for supporting medical students in learning about PP. DeepSeek V3 showed superior accuracy andcompleteness compared to ChatGPT-3.5 and Gemini 2.0 towards all evaluated domains. All models produced almost safe and relevant answers, but the depth and wide-range of clinical content differed significantly. This variation highlights the relevance of relevant model selection for educational purposes. In addition, these findings suggest that, when paired with appropriate guidance and critical thinking, advanced AI models in particular those optimized for medical trainees or scholars/professors may present an ideal platform for medical education and teaching support. This is particularly relevant for challenging pediatric topics, including PP. However, their use should not always replace traditional learning methods, techniques, and clinical mentoring.

## Supplementary Information

Supplementary Information 1.

Supplementary Information 2.

## Data Availability

The data generated during the current study are available from the corresponding authors on a reasonable request.
