# Supplementary material for: Comparing ChatGPT-3.5, Gemini 2.0, and DeepSeek V3 for pediatric pneumonia learning in medical students
Source: Sci Rep. 2025 Nov 18;15:40342. doi: 10.1038/s41598-025-27722-2 (PMC12627666; doi:10.1038/s41598-025-27722-2)
Supplement: Supplementary file 1 — Supplementary Information 1. [file 41598_2025_27722_MOESM1_ESM.doc]

**Supplementary Table 1.** List of 27 expert-designed questions on pediatric pneumonia, grouped into five clinical domains (Definition and Clinical Presentation, Etiology, Diagnostics, Complications, Management). These questions were used to evaluate AI model responses for accuracy, completeness, and safety.

| **Definition and Clinical Presentation:**   1. What is pneumonia? 2. Why is it difficult to determine the cause of pneumonia in children? 3. What are the clinical symptoms of pneumonia in infants? 4. What are the symptoms of pneumonia in older children? 5. What are the findings or risk factors that suggest severe pneumonia? |
| --- |
|  |
| **Etiology and Age-Specific Pathogens:**   1. What pathogens most commonly cause pneumonia in children? 2. What is the most common cause of bacterial pneumonia in children? 3. What are the common causes of pneumonia in newborns (<3 weeks)? 4. What are the causes of pneumonia in infants between 3 weeks and 3 months? 5. What are the causes of pneumonia in children between 4 months and 4 years old? 6. What pathogens commonly cause pneumonia in children older than 5 years? |
|  |
| **Diagnostics and Imaging:**   1. How is pneumonia usually diagnosed? 2. What is the role of laboratory tests such as CRP and procalcitonin in pneumonia? 3. Should a chest X-ray be performed in every child suspected of having pneumonia? 4. How do the radiological appearances of viral pneumonia and bacterial pneumonia differ? 5. When to take a blood culture in a child with pneumonia |
|  |
| **Complications**   1. What are the complications of pneumonia? 2. How to understand if parapneumonic pleural effusion occurs due to pneumonia and what to do in diagnosis |
|  |
| **Management, Treatment and Prevention:**   1. In what cases should a child with pneumonia be hospitalized? 2. When should a child with pneumonia be admitted to the intensive care unit 3. What is the first choice antibiotic for outpatient treatment of community-acquired pneumonia? 4. What is the initial antibiotic treatment for pneumonia in a hospitalized child? 5. How to treat atypical pneumonia 6. When should attention be paid to particle-borne MRSA infection in pneumonia cases and what is its treatment? 7. How are parapneumonic pleural effusion and empyema treated? 8. How long is the usual duration of antibiotic treatment for pneumonia? 9. What can be done to prevent childhood pneumonia? |
|  |
