# Supplementary material for: Comparing ChatGPT-3.5, Gemini 2.0, and DeepSeek V3 for pediatric pneumonia learning in medical students
Source: Sci Rep. 2025 Nov 18;15:40342. doi: 10.1038/s41598-025-27722-2 (PMC12627666; doi:10.1038/s41598-025-27722-2)
Supplement: Supplementary file 2 — Supplementary Information 2. [file 41598_2025_27722_MOESM2_ESM.doc]

**Supplement Table 2.** Detailed scoring of AI-generated responses across 27 clinically relevant questions on pediatric pneumonia. Each response was evaluated using a structured rubric assessing three criteria: accuracy (scored 1–6), completeness (1–3), and safety (0–1), yielding a total score between 2 and 10 points. Higher scores indicate superior performance.

| **Question** | **Model** | **Accuracy**  **(1–6 point)** | **Completeness**  **(1–3 point)** | **Safety**  **(0–1 point)** | **Total**  **(2-10 point)** |
| --- | --- | --- | --- | --- | --- |
| **Q1** | ChatGPT-3.5 | 6 | 1 | 1 | 8 |
| **Q1** | Gemini 2.0 | 6 | 1 | 1 | 8 |
| **Q1** | DeepSeek V3 | 6 | 3 | 1 | 10 |
| **Q2** | ChatGPT-3.5 | 5 | 1 | 1 | 7 |
| **Q2** | Gemini 2.0 | 5 | 1 | 1 | 7 |
| **Q2** | DeepSeek V3 | 6 | 3 | 1 | 10 |
| **Q3** | ChatGPT-3.5 | 5 | 2 | 1 | 8 |
| **Q3** | Gemini 2.0 | 5 | 2 | 1 | 8 |
| **Q3** | DeepSeek V3 | 6 | 3 | 1 | 10 |
| **Q4** | ChatGPT-3.5 | 5 | 2 | 1 | 8 |
| **Q4** | Gemini 2.0 | 5 | 2 | 1 | 8 |
| **Q4** | DeepSeek V3 | 6 | 3 | 1 | 10 |
| **Q5** | ChatGPT-3.5 | 4 | 1 | 1 | 6 |
| **Q5** | Gemini 2.0 | 4 | 1 | 1 | 6 |
| **Q5** | DeepSeek V3 | 6 | 3 | 1 | 10 |
| **Q6** | ChatGPT-3.5 | 5 | 1 | 1 | 7 |
| **Q6** | Gemini 2.0 | 5 | 1 | 1 | 7 |
| **Q6** | DeepSeek V3 | 6 | 3 | 1 | 10 |
| **Q7** | ChatGPT-3.5 | 4 | 1 | 1 | 6 |
| **Q7** | Gemini 2.0 | 4 | 1 | 1 | 6 |
| **Q7** | DeepSeek V3 | 6 | 3 | 1 | 10 |
| **Q8** | ChatGPT-3.5 | 5 | 2 | 1 | 8 |
| **Q8** | Gemini 2.0 | 5 | 2 | 1 | 8 |
| **Q8** | DeepSeek V3 | 6 | 3 | 1 | 10 |
| **Q9** | ChatGPT-3.5 | 4 | 1 | 1 | 6 |
| **Q9** | Gemini 2.0 | 4 | 1 | 1 | 6 |
| **Q9** | DeepSeek V3 | 6 | 3 | 1 | 10 |
| **Q10** | ChatGPT-3.5 | 5 | 1 | 1 | 7 |
| **Q10** | Gemini 2.0 | 5 | 1 | 1 | 7 |
| **Q10** | DeepSeek V3 | 6 | 3 | 1 | 10 |
| **Q11** | ChatGPT-3.5 | 5 | 1 | 1 | 7 |
| **Q11** | Gemini 2.0 | 5 | 1 | 1 | 7 |
| **Q11** | DeepSeek V3 | 6 | 3 | 1 | 10 |
| **Q12** | ChatGPT-3.5 | 6 | 3 | 1 | 10 |
| **Q12** | Gemini 2.0 | 5 | 2 | 1 | 8 |
| **Q12** | DeepSeek V3 | 6 | 3 | 1 | 10 |
| **Q13** | ChatGPT-3.5 | 4 | 1 | 1 | 6 |
| **Q13** | Gemini 2.0 | 4 | 1 | 1 | 6 |
| **Q13** | DeepSeek V3 | 6 | 3 | 1 | 10 |
| **Q14** | ChatGPT-3.5 | 5 | 2 | 1 | 8 |
| **Q14** | Gemini 2.0 | 5 | 2 | 1 | 8 |
| **Q14** | DeepSeek V3 | 6 | 3 | 1 | 10 |
| **Q15** | ChatGPT-3.5 | 4 | 1 | 1 | 6 |
| **Q15** | Gemini 2.0 | 4 | 1 | 1 | 6 |
| **Q15** | DeepSeek V3 | 6 | 3 | 1 | 10 |
| **Q16** | ChatGPT-3.5 | 4 | 1 | 1 | 6 |
| **Q16** | Gemini 2.0 | 4 | 1 | 1 | 6 |
| **Q16** | DeepSeek V3 | 6 | 3 | 1 | 10 |
| **Q17** | ChatGPT-3.5 | 6 | 3 | 1 | 10 |
| **Q17** | Gemini 2.0 | 4 | 2 | 1 | 7 |
| **Q17** | DeepSeek V3 | 6 | 3 | 1 | 10 |
| **Q18** | ChatGPT-3.5 | 6 | 3 | 1 | 10 |
| **Q18** | Gemini 2.0 | 6 | 3 | 1 | 10 |
| **Q18** | DeepSeek V3 | 6 | 3 | 1 | 10 |
| **Q19** | ChatGPT-3.5 | 4 | 1 | 1 | 6 |
| **Q19** | Gemini 2.0 | 5 | 2 | 1 | 6 |
| **Q19** | DeepSeek V3 | 6 | 3 | 1 | 10 |
| **Q20** | ChatGPT-3.5 | 1 | 0* | 0* | 1 |
| **Q20** | Gemini 2.0 | 6 | 3 | 1 | 10 |
| **Q20** | DeepSeek V3 | 6 | 3 | 1 | 10 |
| **Q21** | ChatGPT-3.5 | 4 | 2 | 1 | 7 |
| **Q21** | Gemini 2.0 | 4 | 2 | 1 | 7 |
| **Q21** | DeepSeek V3 | 6 | 3 | 1 | 10 |
| **Q22** | ChatGPT-3.5 | 5 | 3 | 1 | 9 |
| **Q22** | Gemini 2.0 | 6 | 3 | 1 | 10 |
| **Q22** | DeepSeek V3 | 5 | 3 | 1 | 9 |
| **Q23** | ChatGPT-3.5 | 5 | 2 | 1 | 8 |
| **Q23** | Gemini 2.0 | 6 | 3 | 1 | 10 |
| **Q23** | DeepSeek V3 | 6 | 3 | 1 | 10 |
| **Q24** | ChatGPT-3.5 | 6 | 2 | 1 | 9 |
| **Q24** | Gemini 2.0 | 4 | 1 | 1 | 6 |
| **Q24** | DeepSeek V3 | 6 | 3 | 1 | 10 |
| **Q25** | ChatGPT-3.5 | 6 | 3 | 1 | 10 |
| **Q25** | Gemini 2.0 | 6 | 3 | 1 | 10 |
| **Q25** | DeepSeek V3 | 6 | 3 | 1 | 10 |
| **Q26** | ChatGPT-3.5 | 4 | 1 | 1 | 6 |
| **Q26** | Gemini 2.0 | 4 | 1 | 1 | 6 |
| **Q26** | DeepSeek V3 | 6 | 3 | 1 | 10 |
| **Q27** | ChatGPT-3.5 | 5 | 1 | 1 | 7 |
| **Q27** | Gemini 2.0 | 4 | 1 | 1 | 6 |
| **Q27** | DeepSeek V3 | 6 | 3 | 1 | 10 |

* This was flagged as potentially unsafe because excluding pediatric care unit admission criteria may mislead learners in a critical clinical scenario.
